# Seasonal influences on DNA methylation at birth or at sampling time and their interaction with psychosis risk

**DOI:** 10.1093/eep/dvag033

**Published:** 2026-09-23

**Authors:** Ustat Bedi, Ingrid Melle, Ole A Andreassen, Anne Kristin Stavrum, Stephanie Le Hellard

**Affiliations:** Department of Clinical Science, University of Bergen, 5020 Bergen, Norway; Section for Clinical Psychosis Research, Division of Mental Health and Addiction, Oslo University Hospital, 0424 Oslo, Norway; Department of Adult Psychiatry, Institute of Clinical Medicine, University of Oslo, 0372 Oslo, Norway; Centre for Precision Psychiatry, Division of Mental Health and Addiction, Oslo University Hospital and Institute of Clinical Medicine, University of Oslo, 0424 Oslo, Norway; KG Jebsen Centre for Neurodevelopmental Disorders, University of Oslo, 0424 Oslo, Norway; Department of Clinical Science, University of Bergen, 5020 Bergen, Norway; Dr Einar Martens Research Group for Biological Psychiatry, Center for Medical Genetics and Molecular Medicine, Haukeland University Hospital, 5009 Bergen, Norway; Department of Clinical Science, University of Bergen, 5020 Bergen, Norway; Dr Einar Martens Research Group for Biological Psychiatry, Center for Medical Genetics and Molecular Medicine, Haukeland University Hospital, 5009 Bergen, Norway

**Keywords:** EWAS, psychosis, season of birth, season of sampling, DNA methylation

## Abstract

Background: Season at birth has been associated with increased risk for psychotic disorders. Epigenetic markers, which can be influenced by environmental exposures, may help to understand the mechanisms of seasonal effects. Aims: To investigate two types of seasonal influences on DNA methylation: season of sampling and season of birth, and to test whether these seasonal effects interact with psychotic disorder risk. Methods: We used two cohorts: a sample of newborns from the Norwegian Mother, Father, and Child Study (MoBa) and a sample of adult participants from the Thematically Organized Psychosis (TOP) sample. The cosinor function from the R package “seasons” was used to identify seasonally methylated positions (SMPs) associated with season of birth and season of sampling. Meta-analyses were performed between results from the MoBa birth cohort, and the TOP healthy adults samples for both season of birth and season of sampling. Interaction analyses between season of birth/season of sampling and psychosis status were conducted in the TOP cohort. Results: In the meta-analysis of healthy controls from our cohorts, we identified six SMPs (*IQSEC1, CACNA2D3, TMEM86B, CNOT3, ZNF250*, and *ARHGEF3)* and two SMPs (*ZNF250* and *SLC6A19)* associated with season of sampling and season of birth, respectively. In the interaction analysis, one significant SMP (cg13593913,) was found to be associated with a psychosis diagnosis and season of birth. Conclusion: In our study, we show a potential interaction between season of birth and psychosis diagnosis. These findings highlight the importance of considering the season of birth and season of sampling in epigenetic research.

## Introduction

In ancient India, it was believed that the constitution of human beings changes according to the time of year, thus giving rise to different ailments [1]. In human physiology, there are seasonal variations based on the difference in energy consumption throughout the year [2]. In the general population, a peak in body mass index is associated with cold winter months when levels of physical activity are the lowest [3]. Seasonal variation affects multiple physiological processes in humans, including metabolism, physical activity, and immune function. Many infectious, cardiovascular, and psychiatric disorders also show seasonal patterns [4].

Psychiatric illnesses like schizophrenia and bipolar disorder have seasonal variations in their presentation. In a study conducted in Ningbo, China, it was reported that the burden of schizophrenia admission was higher during periods of reduced sunlight versus periods with long sunshine [5]. While seasonal variations are reported in hospital admissions due to psychiatric disorders across the globe, studies have also reported differences in season of birth between patients with psychosis and controls [6–9]. Births in winter months have been associated with increased risk of both schizophrenia and bipolar disorder in the Northern Hemisphere [10,11] while the effect of season of birth is less pronounced in the Southern Hemisphere [11].

The mechanisms underlying the seasonal influence on symptoms or risk of psychosis are not fully understood. Several hypotheses have been proposed, involving environmental factors that vary across seasons, including nutrition, infectious exposure, and socio-cultural factors [12,13]. For example, prenatal maternal infections such as *Toxoplasma Gondii* or genital/reproductive infections have been associated with increased schizophrenia risk in offspring, potentially mediated by elevated maternal third-trimester TNF-α levels [14]. Another study highlighted that this effect might be highest during the second trimester, as this period coincides with the seasonal peak of enterovirus during summer months [15]. Low prenatal vitamin D levels, which are common during winter months due to reduced photoperiod, have also been suggested as a contributing factor [16,17]. In addition, seasonal variation in light exposure may disrupt circadian rhythms, which have been implicated in schizophrenia, bipolar disorder, and depression [18,19]. These hypotheses indicate that seasonal environmental exposures could influence biological pathways relevant to psychosis risk. The mechanism of how this environmental factor may influence the biological risk for illness is not known. Epigenetic variations caused by environmental exposures are hypothesized to reflect nongenetic mechanisms that may affect risk for disorders [20]. An epigenome-wide association study (EWAS) on 27 independent cohorts (*N* = 12 927) from the Pregnancy and childhood epigenetics identified two cytosine–phosphate–guanine sites (CpGs) associated with winter-birth and 26 differentially methylated regions associated with season of birth [21]. While Lockett et al. (2016), found 92 CpGs sites associated with season of birth and allergic outcomes in 18-year-old participants from the Isle of Wight birth cohort (*N* = 367) [22].

Thus, applying a co-sinusoidal method fitted to identify continuous rhythmic variations, we aim to identify seasonal effects on DNA methylation, and also to evaluate whether these effects could interact with schizophrenia or bipolar disorder status [23]. Since the effects in schizophrenia and bipolar disorder are reported as both birth effects and seasonal effects, we are testing three different hypotheses: (i) Are variations in DNA methylation associated with sampling season? (ii) Are variations in DNA methylation associated with the season of birth? (iii) Does the seasonal (birth- or sampling-related) pattern of DNA methylation differ between individuals with and without a psychotic disorder, i.e. is there an interaction between season and psychosis status?

## Results

### Description samples

The TOP sample consists of 1201 healthy controls and 1358 cases with schizophrenia or bipolar disorder. When comparing cases and controls in the cohort, there is a significant difference in the sex proportions (*P* < .02), age (*P* < .001), and sampling year (*P* < .001) between cases and controls (see Table 1). No significant differences between cases and controls were found for the season of birth and season of sampling.

**Table 1:** Socio-demographic characteristics TOP and MoBa samples. The mean birth month in the MoBa sample is similar to the mean birth month in the control sample in the TOP cohort (6.5, SD = 3.5). *Gestational age is reported for MoBa newborns, **Sampling year, ***Sampling month.

	TOP sample			MoBa sample
	Case	Control	*P*-value	Birth cohort
Demographics	1358	1201		4282
Sex, M (% males)	486 (47.4)	631 (52.5)	.02	2193 (51.2)
Age [Mean(SD)]/gestational age*	32.4 (11.2) years	33.9 (9.5) years	.001	39.5 (1.70) months
SYear** [Mean (SD)]	2010.9 (4.8)	2011.7 (4.3)	<.001	2009.5 (211.5)
SMonth*** [Mean (SD)]	6.4 (3.5)	6.7 (3.3)	.164	6.5 (3.5)
Birth month [Mean(SD)]	6.4 (3.4)	6.5 (3.5)	.54	

In the Norwegian Mother, Father, and Child (MoBa) sample (*N* = 4282) 51.2% were male and the mean gestational age was at 39.5 weeks, implying that most of the babies included in the cohort were not prematurely born (see Table 1).

### Seasonal DNA methylation in single cohorts

In the TOP controls, we performed one EWAS for the season of birth and one for the sampling season. In the MoBa birth cohort, since blood is sampled at birth, we performed one EWAS for both the season of sampling and season of birth. No seasonally methylated position (SMP) reach a significant level at false discovery rate (FDR) *q*-value ≤ 0.05 for any of the analyses (see Tables S2–S4).

We compared the lists of nominally significant SMPs of (i) MoBa season of birth/sampling (48 898 SMPs), (ii) TOP season of birth (40 519 SMPs), and (iii) TOP season of sampling (54 053), see Fig. 1. Here, we observed a higher number of SMPs nominally significant for season of sampling in the TOP control sample, and a higher overlap of SMPs between birth/adult for season of sampling (6.7% of total SMPs in birth cohort) compared to the birth/adult season of birth (5.1% of total SMPs in birth cohort).

**Figure 1:**
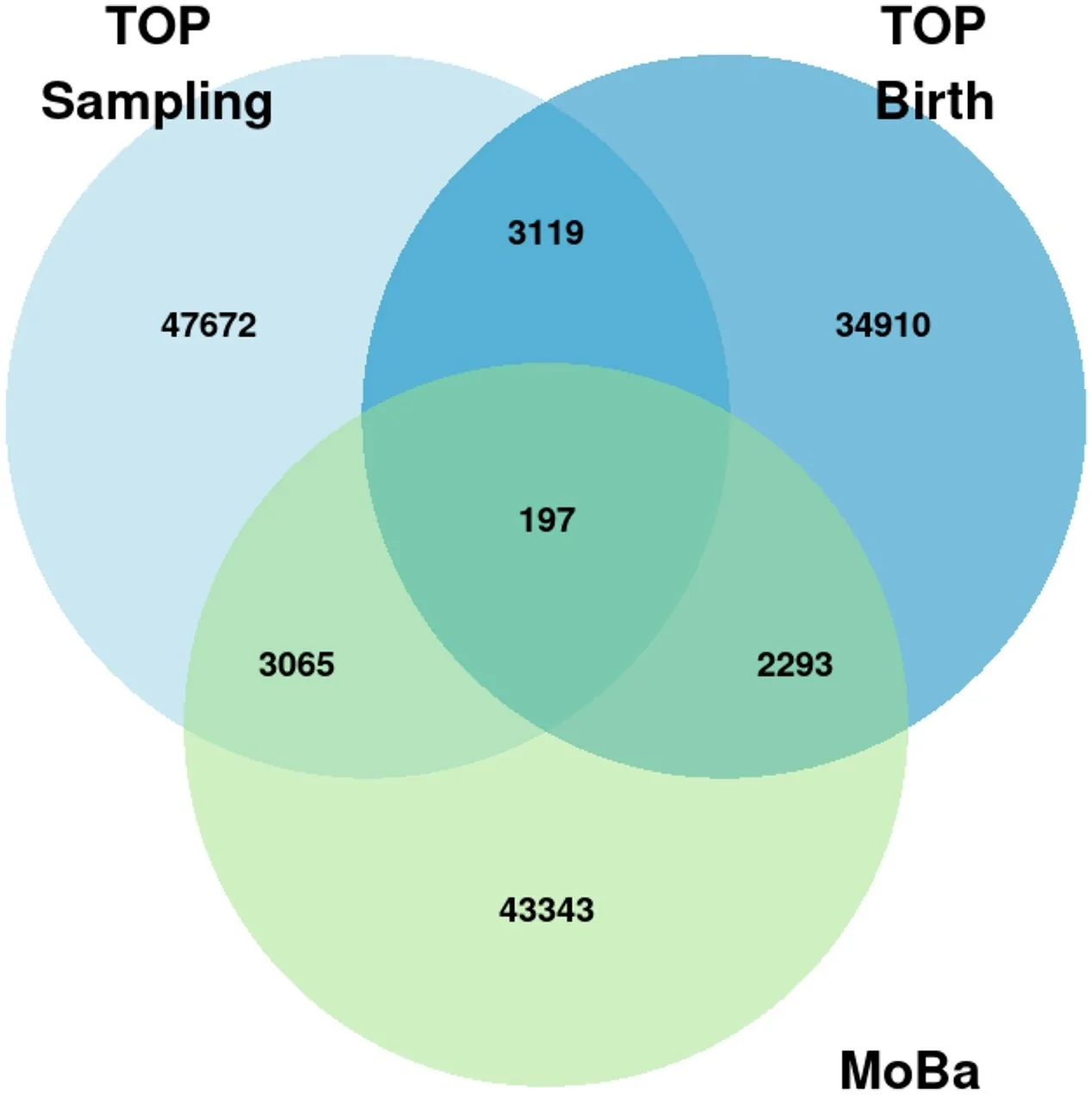
Venn diagram illustrating overlap in CpGs between (i) the MoBa seasonal effects, (ii) TOP season of birth, and (iii) TOP season of sampling. Overlap between SMPs nominally associated with season of birth in adults (TOP Birth), season of sampling in adults (TOP Sampling), and season of birth/sampling in birth cohort (MoBa).

### Meta analysis across birth and adult cohorts

We conducted meta-analyses for (i) season of birth: MoBa birth cohort + TOP controls season of birth and (ii) sample month: MoBa birth cohort + TOP control season of sampling. Two SMPs were significantly associated with birth season for cosinusoidal values. In the meta-analysis for season of sampling, four SMPs were significant for sinusoidal values and two SMPs were significant for cosinusoidal values (FDR *q-*value ≤ 0.05 see Table 2, and Tables S5 and S6). One SMP (cg10639435), located in the gene *ZNF250*, was found to be significantly associated with both birth season and sampling season. A visualization of methylation value (M-values) as a function of the months for cg21408848 in both MoBa and TOP cohort is shown in Fig. S2.

**Table 2:** Significant SMPs identified in meta-analyses (FDR *q*-value ≤ 0.05). ProbeID is the illumina code, SMP position is given based on GRCh38/hg38 built, fdr: flase discovery rate *q*-value, cos: cosinus analysis, and sin: sinus analysis.

		Season of birth meta-analysis (MoBa birth cohort + TOP controls)				
ProbeID	SMP position	Gene	Estimate cos model	fdr_cos	Estimate sin model	fdr_sin
cg10639435	chr8:144 878 837	ZNF250	0.0386	0.0092	−0.000362	0.98
cg09142559	chr5:1 204 003	SLC6A19	0.0446	0.035	−0.00802	0.61
		**Sample month meta-analysis (MoBa Birth cohort + TOP controls)**				
cg21408848	chr3:12 908 349	IQSEC1	0.0172	0.10	0.0470	0.034
cg08362628	chr3:54 827 674	CACNA2D3	0.00834	0.44	−0.0341	0.034
cg20113693	chr19:55 229 389	TMEM86B	−0.000999	0.89	0.0156	0.034
cg21451341	chr19:54 149 599	CNOT3	−0.0117	0.20	0.0325	0.042
cg10639435	chr8:144 878 837	ZNF250	0.0397	0.0013	−0.0062	0.61
cg16848409	chr3:56 858 614	ARHGEF3	0.0322	0.014	−0.00354	0.79

### Interaction season effect with psychosis phenotype

SMPs that showed nominal significance in the meta-analyses were tested for association of an interaction between seasonal effects and case control status in the TOP cohort. One significant SMP (cg13593913, chr 16: 70784939- 70784939, cos *q*-value: 0.049) located in the *VAC14* gene was associated in the interaction between case versus control status and season of birth. As shown on Fig. 2, the pattern of variation in cases versus controls was similar in the two batches of samples from the TOP cohort, with patients showing opposite peaks of methylation compared to controls.

**Figure 2:**
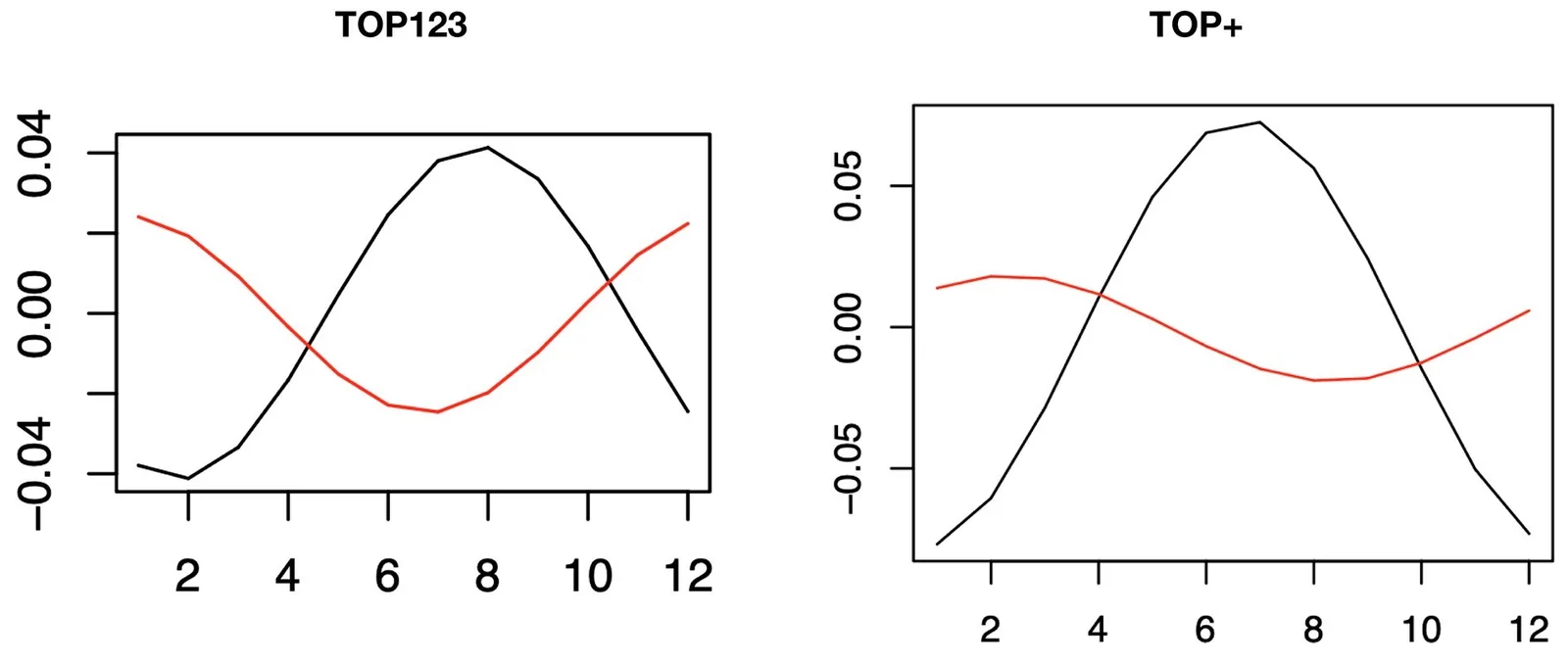
Visualization of the interaction effect on DNA methylation of diagnosis vs no diagnosis by birth season for cg13593913. Fitted curves of M-values variations in cases (black, peaks at 6-8 months) and controls (red with less variations) for the two adult samples are presented with *x*-axis birth month, *y*-axis DNA methylation M-value.

At a less conservative FDR level of *q*-value ≤ 0.25, several SMPs were associated in the interaction analysis, including a SMP located in *MAD1L1* gene, which has been associated with both schizophrenia and bipolar disorder in genetic studies (see Table S7).

No SMPs were significant in the interaction analysis between season of sampling and psychosis (see Table S8).

## Discussion

In this study, we investigated two seasonal influences on DNA methylation that are conceptually and, in the adult cohort, temporally distinct: “season of birth” (a proxy for effect of season environmental exposure at birth) and “season of sampling” (contemporaneous exposure at blood collection). In MoBa, where cord blood is drawn at birth, the two coincide; in TOP, sampled in adulthood, they can be examined independently (using date of birth for “season of birth” and date of blood draw for “season of sampling”).We identified two SMPs associated with season of birth and six SMPs associated with season of sampling. We identified one SMP significantly associated with the interaction between season of birth and psychosis diagnosis and several SMPs with suggestive association in genes previously associated with schizophrenia and bipolar disorder.

The SMP associated with season of birth and diagnosis is located in the *VAC14* gene (VAC14 Component Of PIKFYVE Complex), which encodes a component of a trimolecular complex with *PIKFYVE* (Phosphoinositide Kinase, FYVE-Type Zinc Finger) and *FIG4* (FIG4 Phosphoinositide 5-Phosphatase) [24]. This component tightly regulates the level of phosphatidylinositol 3,5-bisphosphate (PI(3,5)P2), which is critical for survival of neural cells [25]. In a mouse model, where loss of VAC14 protein levels lead to 57% decrease in the levels of PI(3,5)P, this decrease was associated with prenatal death and degeneration in central and peripheral nervous systems [26]. Areas of the brain that were especially affected are pons, medulla and midbrain [26]. In a case report, genetic variants of *VAC14* were found in two unrelated children with onset of progressive basal ganglia degeneration and regression of developmental milestones [27]. Dystonia, nonambulatory, and nonverbal characteristics were noticed in both the children, and striatal abnormalities were noticed on the MRI [27]. Furthermore, in a study looking at 50 families with autosomal recessive parkinsonism, mutations in *VAC14* were reported in patients with progressive and disabling dystonia [24]. Genetic variants in *VAC14* were reported as overlapping genetic risk variants between bipolar disorder and Alzheimer disease [28]. There are no report of association with methylation variations in the literature.

At a more relaxed FDR level (*q*-value ≤ 0.25), an SMP located in *MAD1L1* showed tentative association in the interaction analysis between season of birth and psychosis. Genetic variants located in *MAD1L1* have been associated with schizophrenia and bipolar disorder [29]. Several CpGs located in *MAD1L1* show significantly lower methylation in the superior temporal gyrus and prefrontal cortex of patients with schizophrenia compared to controls [30]. In addition, methylation level of CpGs in *MAD1L1* have been associated with depression and suicide severity in blood and brain tissues [31].

The meta-analysis in healthy controls of the effect of season of sampling found six statistically significant SMPs, located in the genes *ZNF250, IQSEC1, CACNA2D3, TMEM86B, CNOT3, ARHGEF3*, while the meta-analysis of the effect of season of birth found two SMPs located in *ZNF250* and *SLC6A19. ZNF250* (Zinc Finger Protein 250), which is associated with both sampling and birth seasons, has been identified as one of the seven ZNF genes in a cluster conserved in eutherian mammals for at least 130 million years. The most prominent expression of these genes was found in the fetal brain [32]. The genes have not shown association with seasonality or circadian rhythm previously.

The meta-analyses and the overlap analyses comparing the TOP sampling cohort, TOP birth cohort, and the MoBa cohort, identify more effect for season of sampling than for season of birth: (1) more SMPs were significant SMPs for season of sampling in the meta-analyses than for season of birth meta-analysis (6 versus 2, see Table 2); (2) more SMPs were nominally significant for season of sampling than for season of birth in adult cohort (54 053 versus 40 519; see Fig. 1), and (3) the overlap in nominally significant SMPs for season of sampling was higher than for season of birth (3262 vs. 2490; see Fig. 1). Usually season of sampling effects are not corrected for in EWAS and while the effects we identified here are not strong enough to warrant a systematic correction for season of sampling it should be noted that those could affect some analyses that compare cases and controls where season of sampling could be a confounder effect. Notably, one of the significant SMPs for season of sampling has been reported as associated with difference in methylation in the brain of cases with schizophrenia from controls (cg21408848) located in *IQSEC1* [30]. Thus, as we have previously encouraged caution for considering potential time of day effects in biological markers [33], we also now encourage caution for season of sampling effects.

Previous studies have examined the effects of birth season on DNA methylation [21]. In our study, we do not identify the same CpGs to be differentially methylated. Although this could reflect a type 1 error in our study, we argue that this is most likely due to differences in the analytical methods we have used. Here, we have applied a method that has been specifically developed to identify sinusoidal variations in outcome (DNA methylation in that instance), while previous analyses have employed regression methods with categorising seasonal month or season of birth into two or four groups. Seasonal variations are continuous, periodic, and biologically rhythmic, where sinusoidal models, which capture amplitude, phase, and smoothness are better fitted than regression models. Cosinor‐based methods allow precise estimation of peak timing and rhythm parameters [23,15]. Compared to categorical “season” dummy models, sinusoidal models avoid artificial boundaries and overparameterization. Sinusoidal models have been shown empirically to yield good fit for seasonal variations in epidemiology [34] and in gene expression [35] studies.

Interestingly, while sampling season had more effect on DNA methylation in the meta-analysis and overlap analyses, we did not identify interaction effects between season of sampling and psychosis. This suggests that season of birth might have a stronger effect in the interaction with psychosis than season of sampling, or seasonal month of data collection. This might support the observation that season of birth might be an environmental risk factor for psychosis as reported in several studies [10], while monthly variations have been associated with variations in symptom presentations and hospitalizations rather than risk factor to develop psychosis [6–9]. Although several mechanisms, such as prenatal infection [12–14], reduced maternal vitamin D [16,17], or circadian disruption [18,19], have been proposed to explain seasonal variation in psychosis risk, our findings do not directly evaluate these pathways. Instead, the observed seasonal methylation signatures may represent downstream or parallel epigenetic correlates of these broader environmental influences.

We chose to test for an interaction between season of birth and diagnosis, rather than formally testing DNA methylation as a mediator of the season of birth–psychosis association, because our study design cannot support the temporal assumptions mediation analysis requires. Methylation in the TOP cohort was assayed from adult peripheral blood collected at or after diagnosis and following exposure to psychotropic medication known to influence DNA methylation, so we cannot establish that methylation precedes illness onset. Furthermore, our season analyses were performed using the seasonal sine/cosine methods, which have not been adapted for mediation studies. An interaction test does not require this assumption and instead asks whether the seasonal methylation rhythm itself differs between cases and controls. In future studies, especially if longitudinal information is available, mediation analyses would be valuable to establish the possible causal effect of DNA methylation associated with season of birth in psychosis.

Our results should be interpreted considering certain limitations. One of the main limitations of our study is that it is not a longitudinal study, which inhibits us from directly observing how the DNA methylation changes through the seasons. While being relatively large, our sample is limited in size and our findings would warrant replication in larger samples, especially to incorporate sinusoidal models rather than regression/categorical models. Regarding the relevance of the interaction with psychosis, our tissue selection is limited to whole blood and cord blood samples, and not in brain tissues, because of tissue accessibility issues. Even though some research shows overlap between peripheral and brain methylation, to fully transfer our findings into understanding brain function or disease pathology we would need careful validation of our study in brain tissue. This study is also limited to people with European ancestry and specifically focused on a Norwegian cohort. Therefore, the findings need to be reproduced in different cohorts to prove its validity.

## Conclusion

In our study, we investigated how season of sampling and season of birth influence DNA methylation and their interaction with psychosis risk. In the meta-analyses and overlap analyses for the control sample we found six SMPs associated with season of sampling while only two were associated with season of birth. However, in the interaction analyses with psychosis we found one significant SMP associated with the interaction between season of birth and psychosis (*VAC14* gene) while no interaction effects were found between season of sampling and psychosis. Despite our large samples, the effects are small and will warrant replication in other samples.

## Methods

### Cohort information

Birth cohort: The Norwegian Mother, Father, and Child Cohort Study (MoBa) is a nationwide, prospective population-based longitudinal cohort with recruitment conducted between 1999 and 2008. The cohort comprises around 114 500 children, 95 200 mothers, and 75 200 fathers. For children, blood was collected at birth (cord blood). In the present study, four subcohorts of newborns with available DNA methylation data were included: MoBa1 (met001), MoBa2 (met002), MoBa4 (met004), and MoBa8 (met008). Harmonized protocols across subcohorts were used to extract blood from the cord blood collection, and for quality control of the samples, described in Reese *et al*. [36]. The current project received ethical approval from local ethical committee (Regionale komiteer for medisinsk og helsefaglig forskningsetikk—REK #521378). Details on the four cohorts described can be found at (https://github.com/folkehelseinstituttet/mobagen/wiki/Methylation#documentation-valid-for-all-data-sets) [37]. Met001 and met002 comprise cord blood from singleton children, profiled on the Illumina Infinium HumanMethylation450 BeadChip in 2011 and 2013, respectively. Met004 and met008 comprise cord blood from children within mother–father–child triads, profiled on the Illumina Infinium MethylationEPIC BeadChip in 2019 and 2021, only data for the newborns were selected for this study. All participants gave informed consent.

Adult cohort: Patients and heathy controls included in this study are from the Thematically organized psychosis (TOP) sample. Adult participants between the age of 18 and 65 years were recruited in the Oslo area, in Norway, and the cohort has previously been described [38]. We limited our sample to European ancestry as this was the largest subgroup, and to individuals for which DNA methylation was available. This sample included 1201 control and 1358 cases. Diagnoses were given using the structured clinical interview for the Diagnostic and Statistical Manual of Mental Disorders (DSM)-IV Axis 1 disorder (SCID-1) this consisted of 330 individuals with bipolar disorder type I, 201 with bipolar disorder type II, 44 with bipolar disorder not otherwise specified, 77 with schizoaffective disorder, 343 with schizophrenia, and 31 with schizophreniform disorder. In addition, the participants were required to speak a Scandinavian language, have no prior history of head trauma and obtain an IQ score of over 70 [39]. The study was approved by REK (# 2009/2485). All participants gave informed consent.

### DNA methylation data preparation, and quality check

#### MoBa birth cohort

Details about the quality control of the MoBa cohort have previously been specified in Ronningen and Paltiel *et al*. [40]. Quality control was conducted independently for each of the four subcohorts. The minfi package was used to process the raw intensity files (.idat) and generate beta value for each CpG site and detection *P*-value. Between-sample normalization was performed using the Beta Mixture Quantile dilation (BMIQ) method [41].

CpGs missing >10% of methylation data, control probes and nonautosomal probes were removed. Removal of samples with sex mismatch and an average detection of *P*-value across all probes <.05 was conducted. Detailed quality control steps are provided at: (https://github.com/folkehelseinstituttet/mobagen/wiki/Methylation#documentation-valid-for-all-data-sets) [37]. After quality control (QC) the sample remaining for the analyses consisted of 4282 samples (981, 547, 1701, and 1053, for met001, met002, met004, and met0008, respectively).

#### TOP adult cohort

DNA was extracted from peripheral blood samples and the DNA methylation was assayed in four different batches: 1000 samples (2016), 283 samples (2017), and 1082 samples (2019) were assayed using the Illumina Infinium Methylation EPIC array (Illumina, Inc. San Diego), while the last batch with 1089 samples (2024) were assayed on the Infinium® MethylationEPIC BeadChip v2 (Illumina Inc.). The typing was performed at the Life and Brain Genomics GmBH, Bonn, Germany.

The QC and preprocessing of all batches have been described previously. The details of the steps for the first three batches are described in Wortinger *et al*. [42]. Briefly, QC and preprocessing of the data were conducted separately for each batch. Probes and samples that failed QC and sex chromosome probes were removed. Functional normalization was applied to reduce technical variation and to normalize the data. The data from the three batches was then merged, and between batch variation was removed with Combat [43]. This dataset will be referred to as TOP 1–3. The fourth batch of methylation data was preprocessed using an adapted version of the CPACOR pipeline. Probes and samples that failed QC, as well as sex chromosome probes, were removed. The intensity values of the methylated and unmethylated signals were quantile normalized prior to the calculation of beta-values. BMIQ was then applied to the beta-values to make the distribution of the type 2 probes similar to the distribution of type 1 probes. This dataset will be referred to as TOP + . M-values were used for the downstream analyses.

### Association analysis

The samples from MoBa (*N* = 4282) and healthy control samples from TOP1–3 (*N* = 768) and TOP+ (*N* = 326) were used to identify CpGs associated with seasonal patterns. The cosinor method as implemented in the R package “seasons”[44] was used to identify SMPs associated with (1) season of birth and (2) season of sampling. Seasonal variation in DNA methylation was modelled using cosinor regression, implemented in the R package “season” [44]. This method fits sine and cosine terms of calendar month within a generalized linear model to capture periodic (annual) variation in a continuous outcome. For each CpG, M-values were modeled as a function of sin(2πt/12) and cos(2πt/12), where *t* denotes birth month or sampling month as appropriate, together with the covariates specified below. Coefficients and *P*-values were extracted for sine and cosine terms individually, and the resulting nominal *P*-values were corrected for multiple testing across all CpGs using the Benjamini–Hochberg FDR, separately for the cosine and sine terms (fdr_cos and fdr_sin respectively). CpGs reaching FDR *q* ≤ 0.05 for either term were classified as SMPs. The analysis was performed separately for males and females for each of the four MoBa cohorts, TOP1–3 and TOP + . Due to differences in the preprocessing protocols and source of samples (cord blood in MoBa and peripheral blood in TOP), there were also differences in the covariates used in the models. The MoBa cohorts were corrected for gestational age, smoking score, year of birth, four principal components (PCs) from estimated blood cell proportions, two PCs from estimated ancestry, sample processing plate, sentrix position, and control probe PCs. The number of control probe PCs added was decided after evaluating the QQ plots and lambda and varied for the different MoBa data sets (see Table S1). TOP1–3 was corrected for age, smoking score, estimated cell proportions, sampling year, sentrix ID and two genetic PCs. TOP + was corrected for age, smoking score, estimated cell proportions, sampling year, five genetic PCs and five control probe PCs. The TOP cohorts were in addition corrected for season of sampling in the season of birth analysis and corrected for birth month in the analysis of season of sampling. Table S1 provides an overview of the specific models used. SMPs with an FDR <5% was considered significant. Q-Q plots were created to visualize the trend in the SMPs (see, Supplementary 2).

### Overlaps and meta-analyses

The function mixmeta from the R package “mixmeta” [45] was used to summarize the effects from individual analyses. Separate meta-analyses were performed within the cohorts for (i) the MoBa sampling/birth effects, (ii) TOP controls season of birth, and (iii) TOP controls season of sampling. Overlaps were considered between the nominally significant SMPs from (i) MoBa, (ii) TOP controls season of birth, and (iii) TOP controls season of sampling and visualized in a Venn diagram. Meta-analyses across the cohorts were performed to identify common effects associated with season of birth: meta-analysis of MoBa + TOP controls season of birth; and common effects for sampling season: MoBa + TOP controls season of sampling.

### Interaction analysis

Interaction analysis between season of birth/season of sampling with the presence or absence of a psychotic disorder (i.e. cases and controls from the TOP sample) was performed for SMP with a nominally significant *P*-value <.05 in the meta-analyses (season of birth and season of sampling). The interaction analysis was done on the TOP1–3 and TOP+, which contains samples from individuals with psychiatric disorders (schizophrenia = 451, bipolar disorder = 907). The cosinor function was adapted to fit the interaction with season.

## Supplementary Material

dvag033_Supplemental_Files

## Data Availability

The data that support the findings of this study are available upon request, but restrictions apply regarding the availability of these data. Access can only be given after approval by the Norwegian Ethical committees on the grounds that the applications are consistent with the consent provided. Specific questions regarding access for data in this study can be directed to stephanie.lehellard@uib.no
