# Supplementary material for: Seasonal influences on DNA methylation at birth or at sampling time and their interaction with psychosis risk
Source: Environ Epigenet. 2026 Sep 23;12(1):dvag033. doi: 10.1093/eep/dvag033 (PMC13660475; doi:10.1093/eep/dvag033)

Supplementary figure 2. Visualisation of sampling season variations for DNAm in cg21408848 (annotated to IQSEC1) in the 4 MoBa newborn samples and the two TOP controls samples.

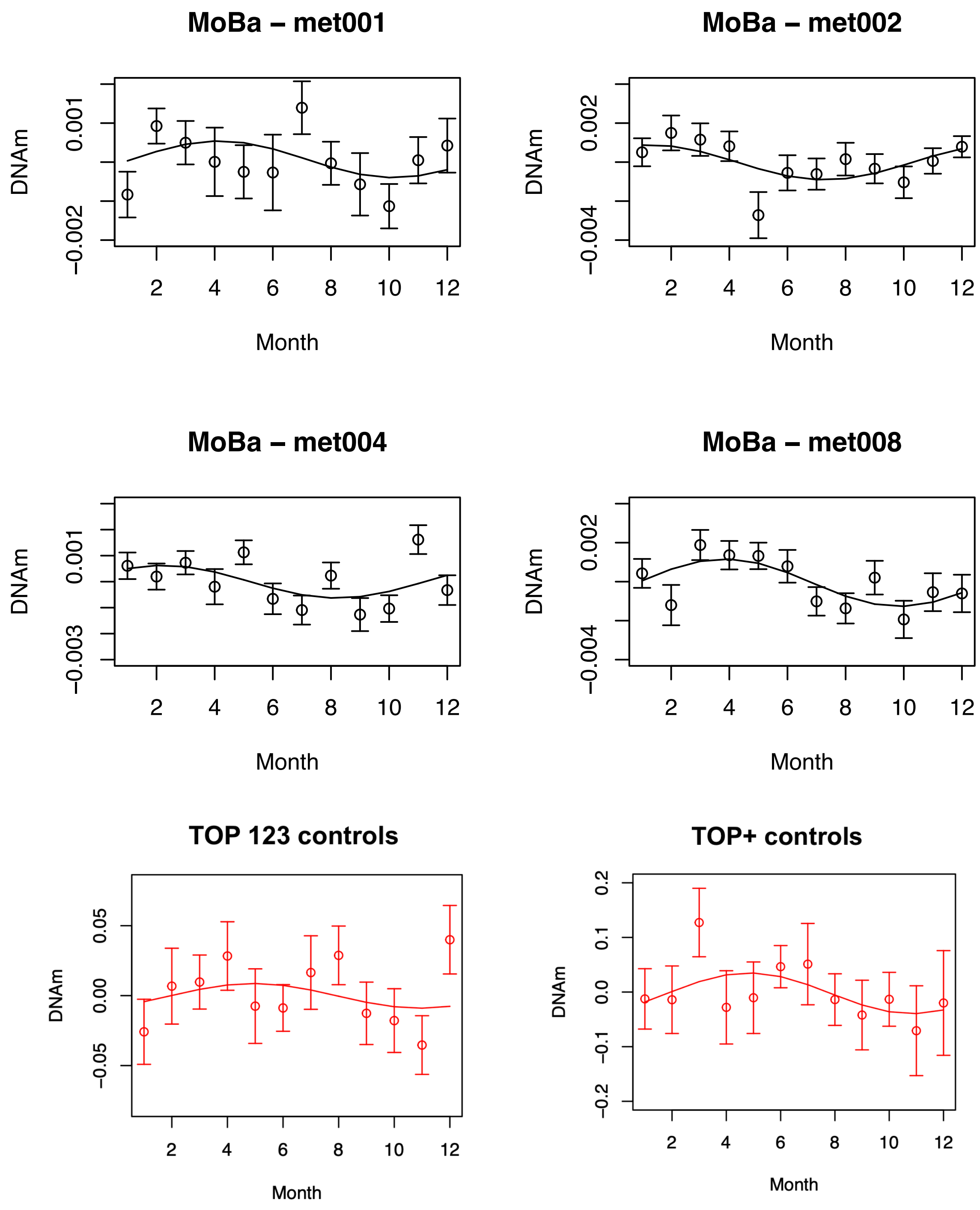

Supplement: Supplementary file 1 [file dvag033_supplemental_files.zip › SuppFig2.pdf]
