# Supplementary material for: Seasonal influences on DNA methylation at birth or at sampling time and their interaction with psychosis risk
Source: Environ Epigenet. 2026 Sep 23;12(1):dvag033. doi: 10.1093/eep/dvag033 (PMC13660475; doi:10.1093/eep/dvag033)
Supplement: Supplementary file 1 [file dvag033_supplemental_files.zip › SuppFigure1_QQplots.pdf]

Supplementary Figure 1. QQ_plots

Meta-Analysis TOP+MoBa Birth Season Cosinus	Meta-Analysis TOP+MoBa Sampling Season Cosinus
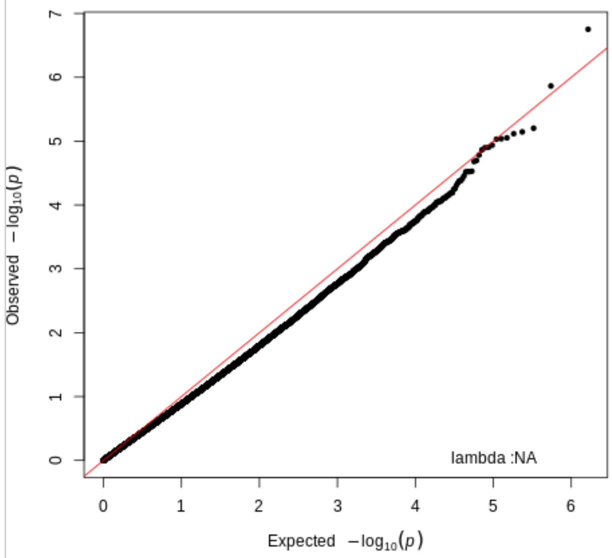	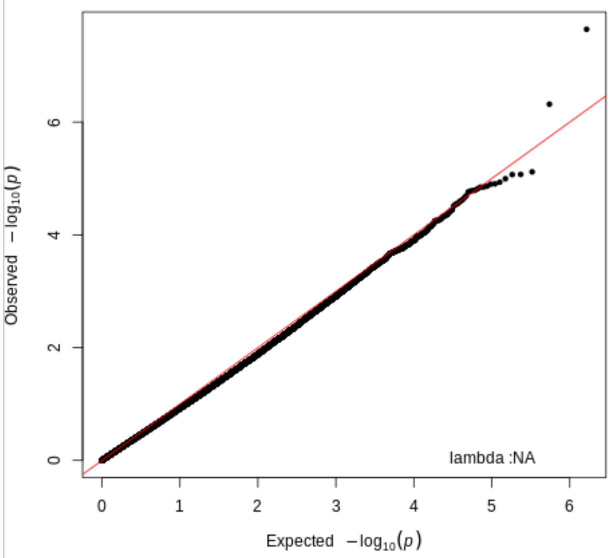
Meta-Analysis TOP+MoBa Birth Season Sinus	Meta-Analysis TOP+MoBa Sampling Season Sinus
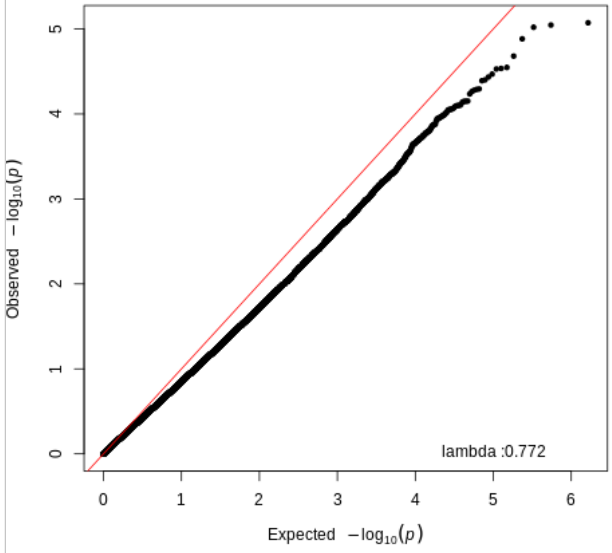	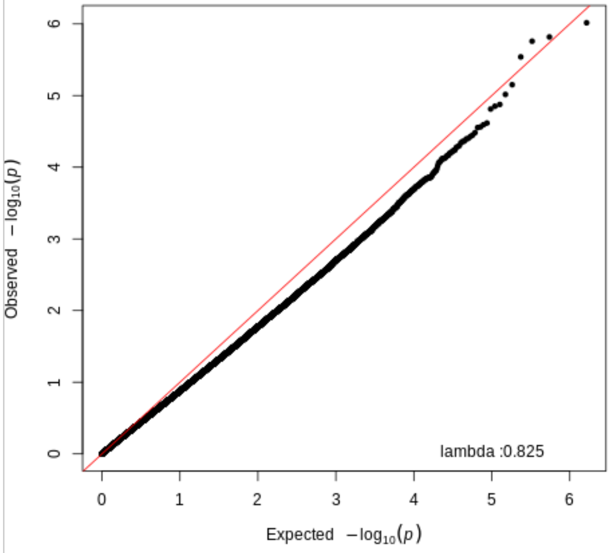
